# Supplementary figures and images for: Immunoglobulin superfamily member 10 is a novel prognostic biomarker for breast cancer
Source: PeerJ. 2020 Oct 21;8:e10128. doi: 10.7717/peerj.10128 (PMC7585383; doi:10.7717/peerj.10128)

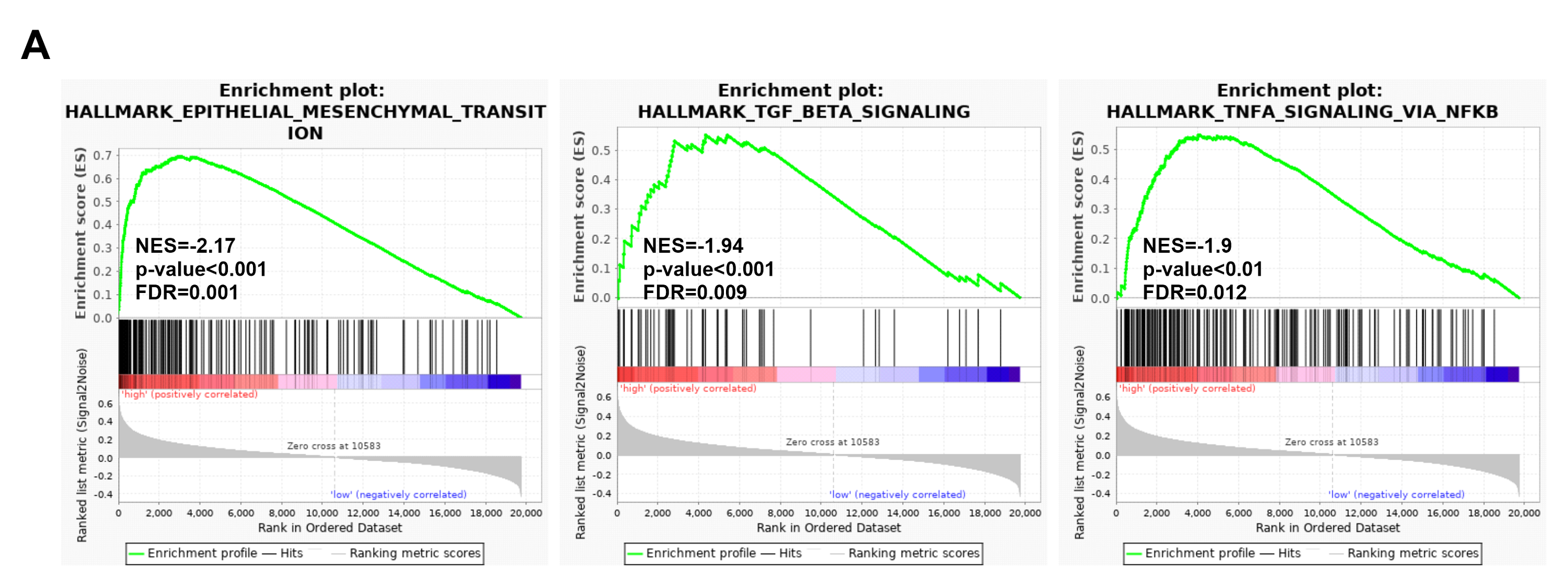

Supplement: Supplemental Information 1 — The gene sets that were significantly associated with IGSF10 with normal P-value < 0.05 and false discovery rate (FDR) < 0.25. Gene sets were ranked by normalized enrichment score NES. (A) The gene sets enriched in the TCGA dataset. [file peerj-08-10128-s001.png]
