# Supplementary material for: Immunoglobulin superfamily member 10 is a novel prognostic biomarker for breast cancer
Source: PeerJ. 2020 Oct 21;8:e10128. doi: 10.7717/peerj.10128 (PMC7585383; doi:10.7717/peerj.10128)
Supplement: Supplemental Information 8 [file peerj-08-10128-s008.doc]

RNA isolation manufacturer's instructions: 1. When precipitating RNA from small sample quantities; 2. Add 0.5 mL of 100% isopropanol to the aqueous phase, per 1 mL of TRIzol® Reagent used for homogenization; 3. Incubate at room temperature for 10 minutes; 4. Centrifuge at 12,000 × g for 10 minutes at 4°C; 5. Proceed to RNA wash; 6. Remove the supernatant from the tube, leaving only the RNA pellet; 7. Wash the pellet, with 1 mL of 75% ethanol per 1 mL of TRIzol® Reagent used in the initial homogenization; 8. Vortex the sample briefly, then centrifuge the tube at 7500 × g for 5 minutes at 4°C. Discard the wash; 9. Vacuum or air dry the RNA pellet for 5–10 minutes. Do not dry the pellet by vacuum centrifuge; 10. Resuspend the RNA pellet in RNase-free water or 0.5% SDS solution (20–50 μL) by passing the solution up and down several times through a pipette tip; 11. Incubate in a water bath or heat block set at 55–60°C for 10–15 minutes; 12. Proceed to downstream application, or store at –70°C.
