# Supplementary material for: Immunoglobulin superfamily member 10 is a novel prognostic biomarker for breast cancer
Source: PeerJ. 2020 Oct 21;8:e10128. doi: 10.7717/peerj.10128 (PMC7585383; doi:10.7717/peerj.10128)
Supplement: Supplemental Information 9 [file peerj-08-10128-s009.pdf]

GENEWIZ®

Solid science. Superior service.

Cell Line Authentication Report

GENEWIZ, Inc. Suzhou

C3 Building, 218 Xinghu Road,

Suzhou Industrial Park, 215123,

Suzhou, China

Fax: 010-59458058

Email: Genomics.China@genewiz.com.cn

www.genewiz.com.cn

GENEWIZ , Inc. Suzhou
C3 Building, 218 Xinghu Road,
Suzhou Industrial Park, 215123,
Suzhou, China

Cell Line Authentication Report

Customer: PengWeiYan

Institution: The First Affiliated Hospital of Chongqing Medical University

Quotation Number: 80-65747945

Completion Date：07/10/2017

1. **Sample ID: MB231**
2. **Original Material: Cell pellets**
3. **Methods:**

1).Genomic DNA was extracted from the cell pellets provided by the customer.

2).Samples, together with positive and negative control were amplified using GenePrint 10 System (Promega).

3). Amplified products were processed using the ABI3730xl Genetic Analyzer.

4).Data were analyzed using GeneMapper4.0 software and then compared with the ATCC, DSMZ or JCRB databases for reference matching.

4. **Results:**

1) 10 Loci STR Profile：

Genetic Site	Customer sample		
(Locus)	MB231		
Amelogenin	X		
CSF1PO	12	13	14
D13S317	13		
D16S539	12		
D5S818	12		
D7S820	8	9	
THO1	7	9.3	
TPOX	8	9	
vWA	15	18	
D21S11	33.2		

GENEWIZ , Inc. Suzhou
C3 Building, 218 Xinghu Road,
Suzhou Industrial Park, 215123,
Suzhou, China

<<<If the Percent match is not 100%, search for reference matching with the ATCC, DSMZ or JCRB databases and add the match results.

Addendum: Comparative output from the ATCC STR Profile database

Result of STR matching analysis by your data.

- DSMZ Profile Database -

A graphical presentation is shown at the bottom of this page.

EV	Cell No.	Cell name	Locus names									Figures
			D5S818	D13S317	D7S820	D16S539	VWA	TH01	AM	TPOX	CSF1PO	
	Query (Your Cell)		12, 12	13, 13	8, 9	12, 12	15, 18	7, 9.3	X, X	8, 9	12, 13, 14	
0.97(36/37)	732	MDA-MB-231	12,12	13,13	8,9	12,12	15,18	7,9.3	X,X	8,9	12,13	-
0.97(36/37)	HTB-26	MDA-MB-231	12,12	13,13	8,9	12,12	15,18	7,9.3	X,X	8,9	12,13	-
0.70(26/37)	CCL-235	SW837 [SW-837]	12,12	13,13	9,12	12,12	15,16	9.3,9.3	X,X	8,9	10,10	-
0.65(24/37)	CRL-7405	Hs 677.Rec	12,12	13,14	9,11	11,12	15,19	6,9.3	X,X	8,9	11,12	-
0.65(24/37)	CRL-7406	Hs 677.Sk	12,12	13,14	9,11	11,12	15,19	6,9.3	X,X	8,9	11,12	-
0.65(24/37)	CRL-7407	Hs 677.St	12,12	13,14	9,11	11,12	15,19	6,9.3	X,X	8,9	11,12	-
0.65(24/37)	CRL-7408	Hs 677.Tg	12,12	13,14	9,11	11,12	15,19	6,9.3	X,X	8,9	11,12	-

>>>

2) Electrophoretogram

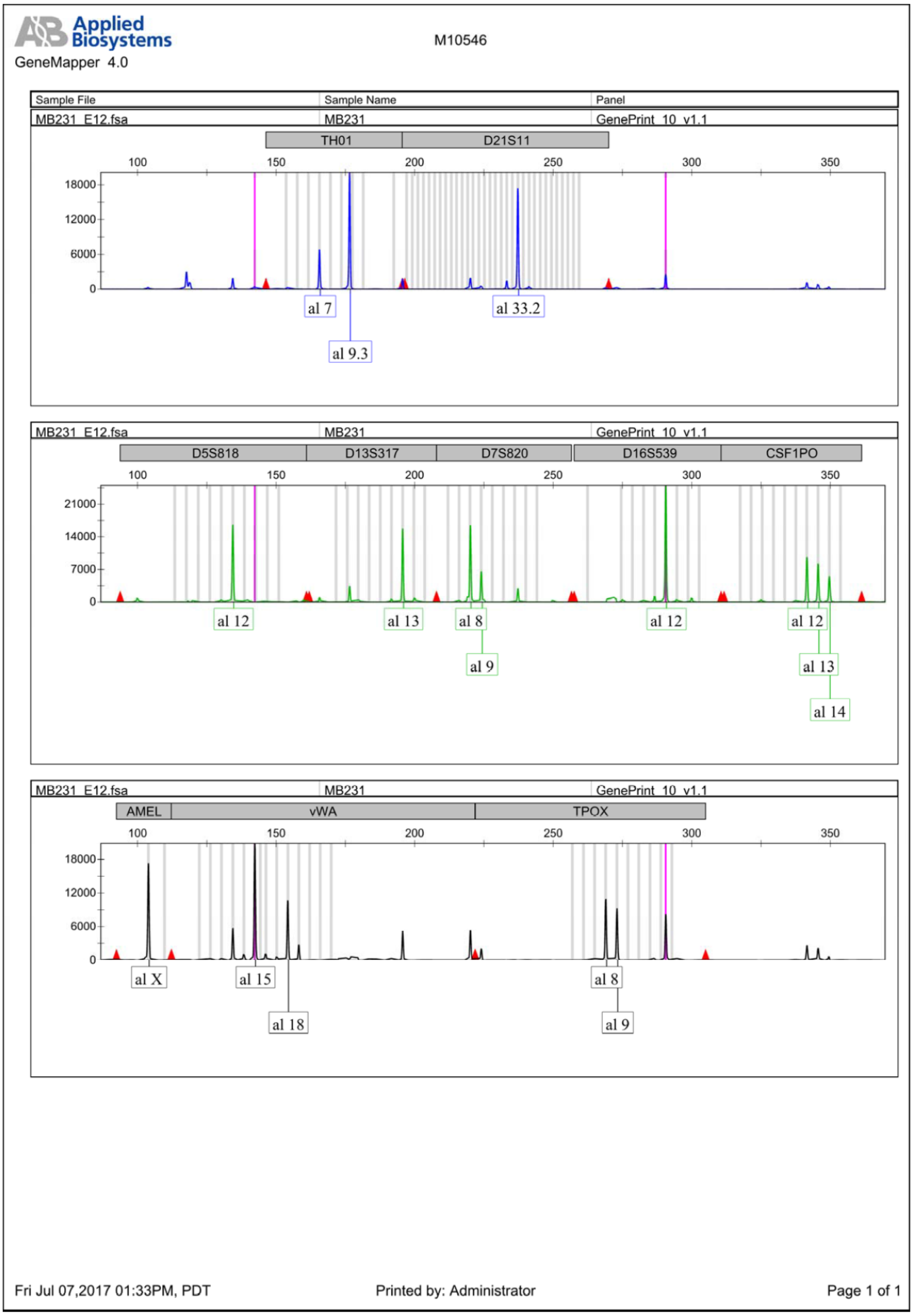

Note： Raw data in appendix
