# Supplementary material for: Immunoglobulin superfamily member 10 is a novel prognostic biomarker for breast cancer
Source: PeerJ. 2020 Oct 21;8:e10128. doi: 10.7717/peerj.10128 (PMC7585383; doi:10.7717/peerj.10128)
Supplement: Supplemental Information 10 [file peerj-08-10128-s010.pdf]

Cell Line Authentication Report

GENEWIZ

C3 Building, 218 Xinghu Road

Suzhou Industrial Park, 215123

Suzhou, China

Tel: 400-8100-669

www.genewiz.com

GENEWIZ, Suzhou
C3 Building, 218 Xinghu Road
Suzhou Industrial Park, 215123
Suzhou, China
+86 400-8100-669
www.genewiz.com.cn

Cell Line Authentication Report

Customer: Weiyan.Peng

Quotation Number: 80-393307609

Completion Date：12/06/2019

1. **Sample ID: MCF7**
2. **Original Material: Cell pellets**
3. **Methods:**

1).Genomic DNA was extracted from the cell pellets provided by the customer.

2).Samples, together with positive and negative control were amplified using GenePrint 10 System (Promega).

3). Amplified products were processed using the ABI3730xl Genetic Analyzer.

4). Data were analyzed using GeneMapper4.0 software and then compared with the ATCC, DSMZ, JCRB and RIKEN etc. databases for reference matching.

4. **Results:**

1) 10 Loci STR Profile：

Genetic Site	Cell Bank information		Customer sample	
(Locus)	MCF7		MCF7	
Amelogenin	X	X	X	X
CSF1PO	10	10	10	10
D13S317	11	11	11	11
D16S539	11	12	11	12
D5S818	11	12	11	12
D7S820	8	9	8	9
THO1	6	6	6	6
TPOX	9	12	9	12
vWA	14	15	14	15
D21S11			30	30
Percent match between the sample and the database profile：100%				

Summary:

Your cell line is considered to be “identical” to the reference cell line in the Cell Bank STR database, as the STR profile yields a 100% match.

GENEWIZ

GENEWIZ, Suzhou
C3 Building, 218 Xinghu Road
Suzhou Industrial Park, 215123
Suzhou, China
+86 400-8100-669

Notes：

1. P=100% x (2xM)/N; M=18, N=36, P=100% x (2x18)/36=100%

M: number of the matching peaks;   N: number of all peaks

2. Based on the ANSI Standard, cell lines with ≥80% match are considered to be related; i.e., derived from a common ancestry. Cell lines with between a 55% to 80% match require further profiling for authentication of relatedness.

3. The short tandem repeat (STR) profile generated by GENEWIZ is indicative only of the sample sent to GENEWIZ at the time it was sent. This data and analysis are for research use only.

GENEWIZ

2) Electrophoretogram

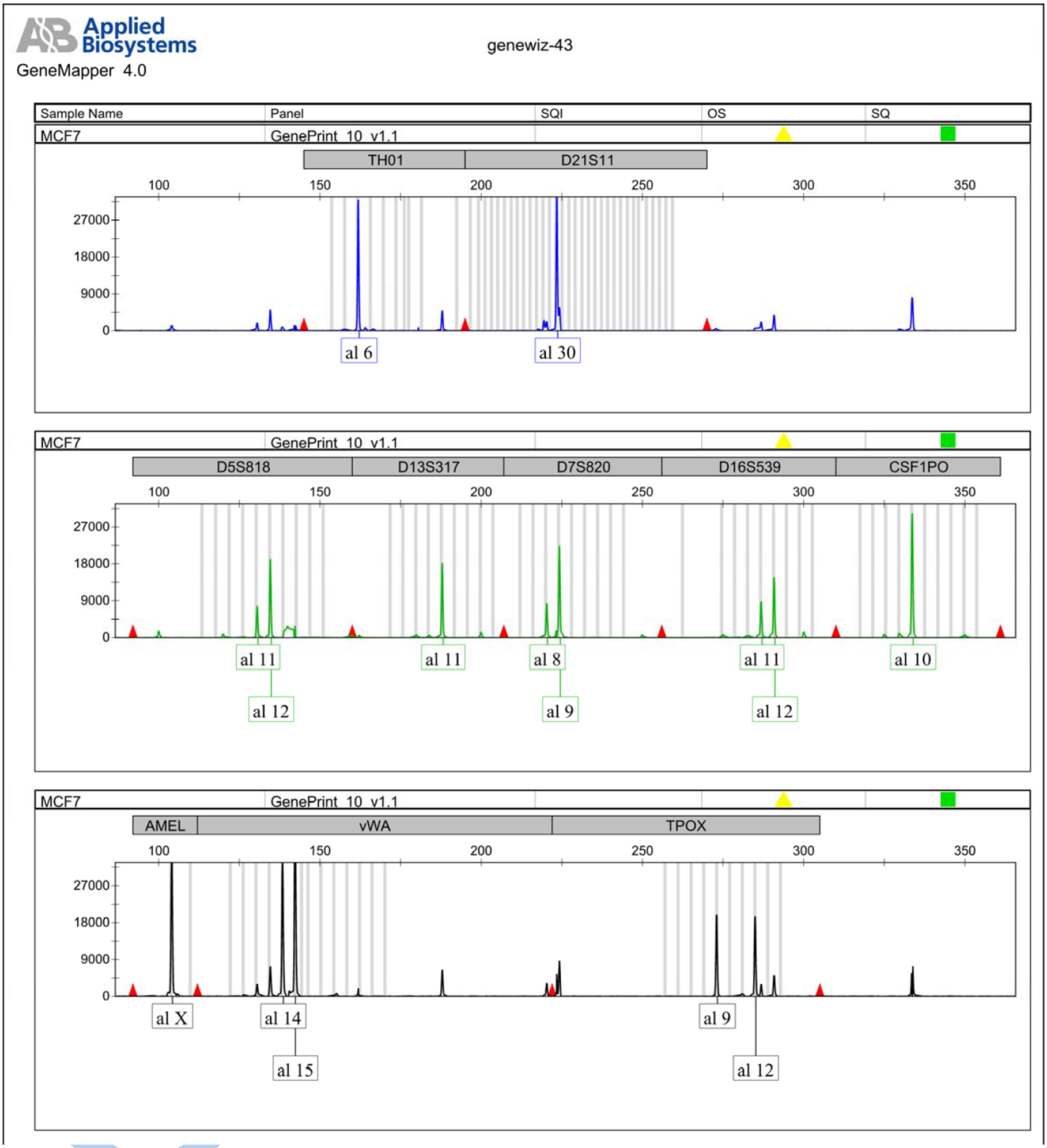

Note： Raw data in appendix
